# Preventive Dentistry*Extract from a paper read by Dr. Tracy before the New York State Dental Society and printed in the “Dental Cosmos” under the title, “The Next Quarter Century in Dentistry.”

**Published:** 1919-05

**Authors:** W. D. Tracy


### PREVENTIVE DENTISTRY*

BY W. D. TRACY, D.D.S.

The dental mind has for many years been applied to the study of prevention of dental diseases, and with certain limitations preventive dentistry is an accomplished fact in the present day.

The systematic prophylactic treatment of children and youthful patients, as well as of adults with restored or cured cases, has proved its value as a preventive measure, and it is now an established part of the work in every well-regulated dental practice.

What has seemed to be the most overwhelming problem faced by the dental profession is the care of the teeth of the millions of school children in this country. The economic waste and the actual cash loss due to ill health and mental deficiency caused solely by dental disease and defects in all large municipalities is too well recognized to need reiteration.

While preventive dentistry is applicable to patients of all ages, its most important field of usefulness is perhaps in its application to the school children, and in several sections of the United States the systematic prophylactic treatment of public school children has been carried on with the most salutary results. Preventive dentistry as applied to the school children of our country is really in its infancy, but the present indications warrant the prophecy that during the next twenty-five years the development in this special field of work will be so great that every school in the country will be obliged by law to provide prophylactic treatment by properly trained dental hygienists for the children under its control.

* Extract from a paper read by Dr. Tracy before the New York State Dental Society and printed in the "Dental Cosmos" under the title, "The Next Quarter Century in Dentistry."

It is quite possible that in isolated sections where the population is scattered, rural automobile dental clinics will make their periodical rounds under state control to minister to the dental needs of the school children and others who can not go to the larger towns for treatment. This plan has already been initiated in Vermont, and possibly in other states.

In view of the important work done by Dr. Alfred C. Fones of Bridgeport in making preventive dentistry in the schools of his city a reality, and because of his years of study in this and allied problems, his opinion is worthy of record and is quoted as follows: "It is much easier to prophesy the result that may be attained in preventive dentistry in a quarter of a century than to attempt to outline the probable stages through which it will pass in securing that ultimate result. The efforts of our profession will be concentrated upon the prevention of dental caries and pericemental infection from the gingival border. The two great factors that will control these diseases are proper food and prophylaxis."

The dentist will become an educator instructing his patients in matters of diet conducive to normal mouth conditions and bodily health. His greatest field for service will be among the mothers of small children who will learn of the practical elimination of free sugars, white breads, crackers, etc., and that the substitution of simple, wholesome, hard foods will help to produce the ideal arch development and reduce the tendency to dental diseases. The beginning of this education of the public as to proper foods is made practical, even at the present time, by the war situation, which has forcibly eliminated the use of a good part of the free sugars and white breads from the diet. Until this slow educational campaign has made itself felt, the most rigid system of prophylaxis and habitual daily mouth cleanliness must be carried out to overcome the evils of our present form of diet.

The medical profession, as well as the general public, is slowly realizing the great importance of sound teeth and healthy mouths to general health, and this knowledge will become so widespread that a national department of health, of which dentistry will be a very important part, will be created to help produce and maintain health. The value of prevention over cure is already realized to such an extent that every health activity is being carried on along lines that tend to prevent disease.

The national department of health with its own secretarial bureau will find it within its power to compel scientific feeding and care of the body by enforcing the pure food laws and restricting the manufacture of candies, confectionery, etc. The necessity for this restriction is made apparent by the statistics secured in the examination of thousands of mouths of men drafted into the national army. These show that the Italians, with a national consumption of but thirteen pounds of sugar per capita per annum, have teeth in which dental caries is the exception rather than the rule, in contrast to the mouths of the boys of America, whose average consumption of free sugar is about ninety pounds per capita per annum.

It is my belief that the great educational and preventive work will be carried on by an immense corps of dental hygienists in public schools, hospitals, and other public and private institutions, aside from those employed in private practice. The educational and preventive dental clinic for public schools will be universally adopted, and although municipalities may conduct reparative dental clinics, they will be separate and apart from the school buildings. In 1943 fully 30 per cent. of the school children will be free from dental caries, and many of the diseases of childhood will have been stamped out. A toothache will be generally understood as a calamity, as it will usually mean pulp involvement, and for the masses this will mean extraction. Focal infection of the teeth will have long since been acknowledged as an important source of systemic infections, and the dental practitioner will be obliged to be a medically educated man in order that he may have a greater knowledge and broader vision of dentistry, which will be recognized as the most important specialty of medicine.

In spite of all the preventive dentistry that can be accomplished during the next twenty-five years, the profession at large will still be constantly occupied with curative and reparative work in the mouths of people who did not come under the influence of compulsory dentistry as children, or who were personally neglectful of their mouths and teeth after reaching adolescence.

We naturally ask, "What will be considered good practice twenty-five years hence in dealing with a tooth in which the pulp has lost its vitality or which suffers an exposure of the pulp through the removal of deep-seated caries?"

The idealist would say that if all the plans advocated by the champions of preventive dentistry could be put into effect at once and carried out systematically through the next quarter century, there would be no exposed pulps, and therefore no problem in connection with devitalized or infected teeth.

